# The effects of ovarian cancer cell-derived exosomes on vascular endothelial growth factor expression in endothelial cells

**DOI:** 10.17179/excli2019-1800

**Published:** 2019-10-09

**Authors:** Mohammad Ghorbanian, Sadegh Babashah, Farangis Ataei

**Affiliations:** 1 Department of Molecular Genetics, Faculty of Biological Sciences, Tarbiat Modares University, Tehran, Iran; 2 Department of Biochemistry, Faculty of Biological Sciences, Tarbiat Modares University, Tehran, Iran

**Keywords:** ovarian cancer, cancer cell-derived exosomes, Exo-OVCAR-3, vascular endothelial growth factor

## Abstract

Ovarian carcinoma is considered as a major clinical challenge worldwide. Exosomes, nano-sized intraluminal vesicles of multivesicular bodies, are secreted by most types of cells and play an important role in intercellular communication. Cancer cell-derived exosomes can develop cancer progression and metastasis by manipulating the local and distant biological environments. Angiogenesis is an important contributor to tumor progression. Vascular endothelial growth factor (VEGF) is the most potent pro-angiogenic protein and induces proliferation, sprouting, and vessel formation by endothelial cells. In this study, exosomes derived from ovarian epithelial cancer cells OVACAR-3 (exo-OVCAR-3) were successfully isolated and characterized by scanning electron microscopy in terms of size and morphology. Cellular internalization of exosomes labeled with PKH fluorescent dye was monitored by a fluorescence microscope. Our results elucidated that exosomes treatment (100 µg/ml) had a promoting effect on VEGF expression and secretion in endothelial cells. Furthermore, we demonstrated that exo-OVCAR-3 caused an increase in the proliferation and migration rate of endothelial cells. It seems that inducing VEGF by exo-OVCAR-3 can influence the vascular behavior of endothelial cells *in vitro*.

## Introduction

The microenvironment of tumor cells plays a major role in tumor growth; as well as it takes a center stage in regulating some processes such as angiogenesis through remodeling the tissue structure by altering stroma and producing growth factors (Chowdhury et al., 2015[7]). Tumor angiogenesis, forming blood capillaries, is an essential process causing cancer growth. Expression of pro-angiogenic factors such as vascular endothelial growth factor (VEGF) can promote tumors to progress (Lee et al., 2013[19]; Shahi and Pineda, 2008[29]; Welti et al., 2013[33]). The dynamic interaction between the tumor and its microenvironment leads to metastasis which is known as a complex process. Therefore, the formation of a vascular network triggered by angiogenesis causes tumor growth and metastasis (Egeblad et al., 2010[10]; Hanahan and Weinberg, 2011[14]).

Exosomes are a kind of nano-sized extracellular vesicles that can be secreted by most types of cells (Dinkins et al., 2014[8]; Motavaf et al., 2016[24]). Exosomes contain major RNA molecules including mRNAs and microRNAs that are capable of being shuttled from donor cells to recipients (Lee et al., 2013[19]; Pakravan et al., 2017[27]). The significant functions of exosomes in transformation of adjacent cells contributing to tumor cell proliferation, migration, and induction of angiogenesis have been recently demonstrated (Wu et al., 2016[34]).

Epithelial ovarian cancer is the seventh most prevalent type of cancer among females and also the most lethal form of gynecological malignancy in the Western population (Mariappan et al., 2017[22]). Ovarian cancer is often diagnosed late and it is a reason for low survival rate (Liang et al., 2013[20]). In other words, most women diagnosed in the late stage of ovarian cancer have the overall five-year survival rate of approximately 40 % (Banerjee and Kaye, 2013[2]). Different gene expression patterns and the heterogeneity of ovarian cancer mostly suggest that targeted therapy, which involves the use of therapeutic molecules that would specifically modulate the pathways implicated in tumor progression, may be effective only in some patients. Thus, different aspects of targeted therapy and personalized medicine should be further addressed (Wei et al., 2013[32]). 

One of the main processes contributing to cancer development is intercellular communication in the tumor microenvironment. Although intermediary role of extracellular vesicles, exosomes in particular, has been widely investigated, much should be applied to recognize this process thoroughly (Sharma et al., 2017[30]). In the present study, we aimed to investigate the possible paracrine effects of exosomes derived from ovarian epithelial cancer cells OVACAR-3 (exo-OVCAR-3) on proliferation and migration of endothelial cells, especially regarding VEGF expression.

## Materials and Methods

### Cell culture 

The human ovarian epithelial cancer cells (OVACAR-3) and human umbilical vein endothelial cells (HUVECs) were acquired from the Pasteur Institute of Iran, Tehran, Iran. The cells were cultured in Dulbecco's Modified Eagle's Medium (DMEM) supplemented with 10 % heat-inactivated fetal bovine serum (FBS) and 2 mM L-glutamine, and 1 % antibiotics agents (100 U/ml of penicillin and 100 µg/ml of streptomycin) (Gibco BRL, USA); the cells were kept at 37 °C in a humidified atmosphere containing 5 % CO_2_.

### Isolation of exosomes

Ovarian epithelial cancer cells OVACAR-3 were cultured and the supernatant was harvested and stored. Exo-OVCAR-3 were isolated by ExoQuick-TC^TM^ (System Bioscience, USA) according to the manufacturer's instructions. In brief, supernatant derived from cell cultures was centrifuged at 300 g for 15 min and consequently, the cell debris was eliminated. ExoQuick-TC^TM^ Exosome Precipitation Solution was added to the supernatant and the derived solution was refrigerated overnight and then centrifuged at 15,000 g for 30 min. We discarded the supernatant and resuspended the pellets to PBS solution. Finally, the solution was centrifuged at 1,500 g for 5 min to remove the supernatant. The exosome pellets were resuspended in PBS and stored at -20 °C. 

### Scanning electron microscopy 

The purified exosomes were fixed in 2.5 % glutaraldehyde and dehydrated using grading ethanol series. The exosome-containing sample was vacuum-dried on a glass substrate sputter and coated with gold. Eventually, exosome size and morphology were monitored using a scanning electron microscope (Digital SEM, KYKY-EM3200, China). 

### Expression analysis by RT-qPCR

Total RNA was isolated from HUVECs using RiboX reagent following the manufacturer's recommendations and treated with RNase-free DNase (Fermentas, Lithuania). Complementary DNA (cDNA) synthesis was performed using 1 μg of total RNA as a template as well as random hexamers and PrimeScript reverse transcriptase (TAKARA, Japan). Then, the quantitative real-time PCR (RT-qPCR) was performed in an ABI Step One Sequence Detection System (Applied Biosystems, USA) using SYBR^®^ Premix Taq™ II (TAKARA, Japan). The relative expression of each gene was determined using the 2^-ΔΔCt^ method (Hayat Nosaeid et al., 2009[16]; Livak and Schmittgen, 2001[21]). Primer sequences used in RT-qPCR were as follows: VEGFA: forward: 5'-GCAGAAGGAGGAGGGCAGAATCA-3', reverse: 5'-CACACACTCCAGGCCCTCGTC-3'; NFκB1: forward: 5'-TACTCTGGCGCAGAAATTAGGTC-3', reverse: 5'-ACTGTCTCGGAGCTCGTCTATTTG-3', and GAPDH: forward: 5'-CCGAGCCACATCGCTCAG-3', reverse: 5'-GGCAACAATATCCACTTTACCAG-3'.

### Western blotting

To perform Western blot analysis, the equal amount of proteins was drawn out using a lysis buffer (50 mM of Tris-HCl in pH 8.0, 150 mM of NaCl, 2 mM of EDTA and 0.1 % NP-40). This buffer contained a protease inhibitor cocktail (Roche, Switzerland). The proteins were detached on 12 % SDS-PAGE and then transferred onto polyvinylidene difluoride (PVDF) membranes. The membranes floated in blocking solution and were incubated with particular primary antibodies and horseradish peroxidase-conjugated to secondary antibodies. Finally, the protein was exposed to chemiluminescence. 

### Cell proliferation assay

Approximately 5⨯10^4 ^cells in a 24-well plate were seeded and incubated with 100 µg/ml of exo-OVCAR-3 or control (phosphate-buffered saline, PBS). After 24 h and 48 h treatment with exo-OVCAR-3, total cell number was calculated in duplicate by trypan blue exclusion.

### Wound healing assay

To measure cell migration, HUVECs were grown to 80 % confluency in a 24-well plate. After 24 h, the cells were wounded by scratching applied by a 200-ml pipette tip. Then, HUVECs were treated with 100 µg/ml of exo-OVCAR-3 or carrier control (PBS) for 24 h and 48 h. Migration of endothelial cells was quantified and monitored over 24 h and 48 h. For further evaluation, the images were processed by WimScratch (Wound Healing Assay Image Analysis) (https://mywim.wimasis.com).

### Labeling and internalization of exosomes

Exo-OVCAR-3 were labeled using PKH26 red fluorescent labeling kit (Sigma, USA) according to the manufacturer's instructions. In brief, 2 μl of PKH26 was added to 25 μg of exo-OVCAR-3 in 1 ml of diluent C supplied with the kit and incubated for 20 min at room temperature. The exosome-free solution was used as a negative control to investigate the transportation of PKH26 dye. To stop the labeling process, an equal volume of BSA was added, after which the solution was incubated with 18 ml of PBS. Exosomes were re-purified by using the ExoQuick-TC Exosome Precipitation Solution. Eventually, the pellet containing PKH26-labeled exosomes was achieved and the labeled exosomes were incubated with HUVECs at 37 °C with 5 % CO_2_. After 12 h, the cells were washed twice with PBS and prepared for fluorescence uptake monitoring using an inverted fluorescence microscope (Olympus, CKX41).

### ELISA measured VEGF secretion

VEGF secretion was evaluated on the supernatant of endothelial cells which were stimulated by 100 μg/ml of exo-OVCAR-3 or vehicle control (PBS) for 24 h and 48 h. This evaluation was applied using a human VEGF enzyme-linked immunosorbent assay (ELISA) Kit (Abcam, ab100662).

### Statistical analysis

All data are presented as the mean ± standard deviation (SD). All the experiments were performed duplicate or triplicate and the data were analyzed by using Student's *t*-test. *P*-value less than 0.05 was considered statistically significant.

## Results

### Exo-OVCAR-3 isolation and characterization

**Figure 1:**
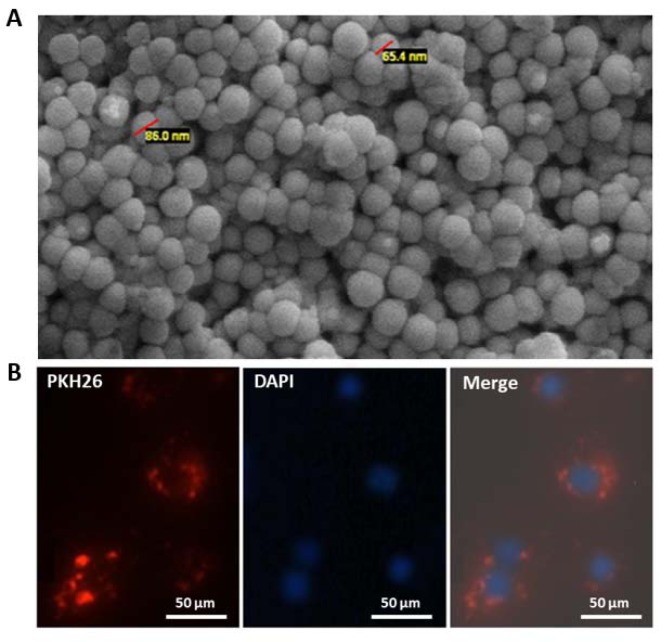
Characterization and cellular uptake of ovarian cancer cell-derived exosomes. (A) Scanning electron micrograph of purified exosomes derived from ovarian cancer cells OVACAR-3 (exo-OVCAR-3) showed isolated vesicles are spherical in shape and around 30-100 nm in size. (B) Cellular uptake of exo-OVCAR-3 labeled with fluorescent red dye PKH26 by endothelial cells. A red fluorescence in the cytoplasm of the HUVECs indicates cellular internalization of exo-OVCAR-3 into HUVECs. DAPI staining was used to visualize nuclei. Bar represents 50 µm.

To investigate the effects of the exosomes secreted by OVACAR-3 ovarian cancer cells on behaviors of endothelial cells, exosomes were isolated from the culture supernatant of OVACAR-3 cells. SEM verified that all the purified exosomes were spherical in shape with a diameter of ~30-100 nm (Figure 1A(Fig. 1)).

### Cellular internalization of exo-OVCAR-3 by endothelial cells

To determine the capacity of exosomes to be transferred to recipient endothelial cells, exo-OVCAR-3 were labeled with the fluorescent dye PKH26 and incubated with HUVECs for 12 h and subsequently cellular uptake of exo-OVCAR-3 was observed under the fluorescence microscopy. We found that PKH26-labeled exosomes were localized in the cytoplasm of HUVECs, implying that exo-OVCAR-3 can be internalized by endothelial cells (Figure 1B(Fig. 1)).

### Exo-OVCAR-3 has a positive effect on VEGF expression in endothelial cells

**Figure 2:**
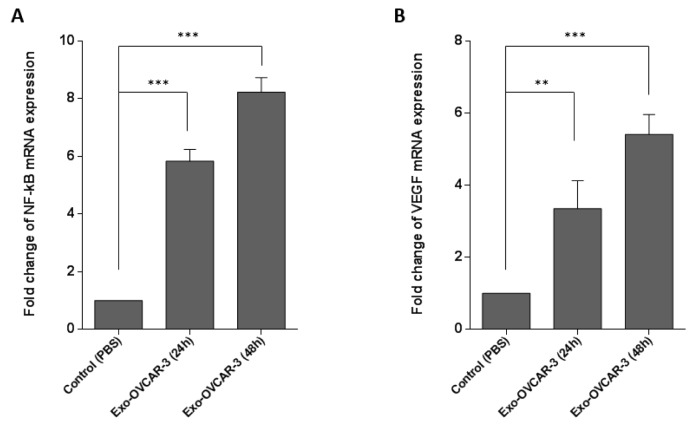
The promoting effects of exosome treatment on VEGF (A) and NF-κB (B) mRNA expression levels. Expression levels assessed by RT-qPCR and normalized to GAPDH as a housekeeping gene in OVACAR-3 cells, after 24 h and 48 h treatment with exo-OVCAR-3 or vehicle control PBS. Columns represent means of three different experiments; bars represent SD.

**Figure 3:**
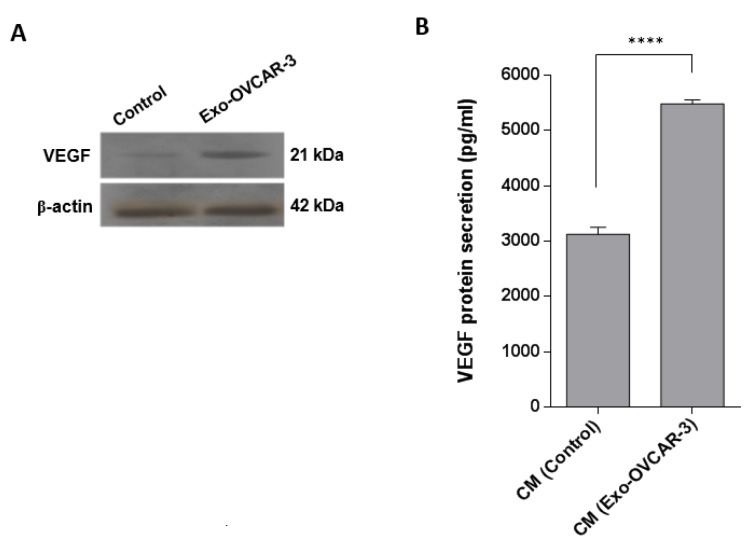
Increasing the VEGF protein level and secretion in/from HUVECs after incubation with exo-OVCAR-3. A) Western blot results showed an increase in VEGF protein levels in endothelial cells 4 h after treatment with exo-OVCAR-3 or PBS. B) ELISA analysis showed that endothelial cells have a significant effect on VEGF secretion 48 h after treatment with exo-OVCAR-3; there was an increase in the amount of VEGF protein secreted into the conditioned media (CM) of HUVECs which was incubated with 100 µg/ml exo-OVCAR-3 than those cells treated with PBS (control group). Columns represent means of three different experiments; bars represent SD.

The vital role of VEGF in angiogenesis has been well documented in previous studies (McMahon, 2000[23]; Wu et al., 2006[35]). It has been also demonstrated that the activation of NF-κB pathway leads to increasing VEGF expression (Bancroft et al., 2001[1]). Thus, to investigate the angiogenic effects of exo-OVCAR-3 on endothelial cells, we evaluated NF-κB and VEGF mRNA expression levels by RT-qPCR, relative to GAPDH as the housekeeping gene, in HUVECs following treatment with exo-OVCAR-3 (100 µg/ml) or vehicle control (PBS). This study showed that exo-OVCAR-3 can up-regulate transcript levels of NF-κB and VEGF in HUVECs in a time-dependent manner (Figure 2(Fig. 2)). Consistently, exo-OVCAR-3 had also a significant influence on VEGF protein levels in HUVECs (Figure 3A(Fig. 3)). Moreover, this study also revealed an increase in VEGF protein secretion of endothelial cells treated with 100 µg/ml of exosomes after 48 h (Figure 3B(Fig. 3)). Thus, the increased VEGF protein expression and secretion after exo-OVCAR-3 treatment was consistent with its augmented mRNA expression levels.

### Exo-OVCAR-3 stimulate HUVECs proliferation and migration 

**Figure 4:**
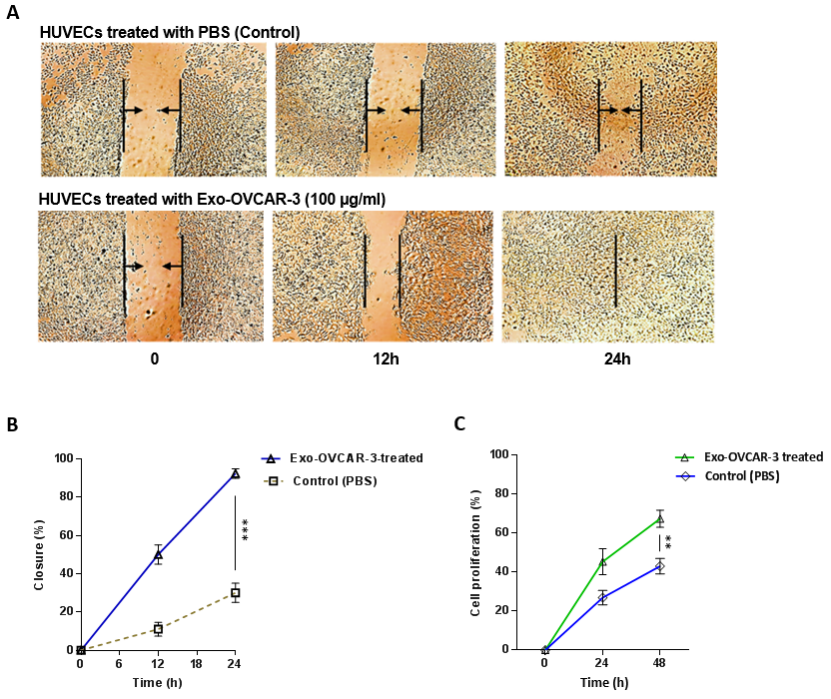
Exo-OVCAR-3 promote the proliferation and migration of HUVECs *in vitro*. A) Representative photomicrographs of the migration of HUVECs treated with exo-OVCAR-3 (100 µg/ml) or PBS (control) at the time of scratch (0 h) and 12 h and 24 h thereafter. B) Analysis of the area covered by cells at the time of scratch (0 h) and 12 h and 24 h thereafter via WimScratch showed a higher migration rate of endothelial cells treated with exo-OVCAR-3 (100 µg/ml) than those cells treated with PBS (control group). C) Exo-OVCAR-3 (100 µg/ml) treatment increased the proliferation rate of HUVECs in a time-dependent manner. Points represent mean of three experiments; bars represent SD.

Previous studies have highlighted an important role of angiogenic VEGF overexpressed in several types of cancers (Borgström et al., 1996[4]; Dvorak et al., 1999[9]). VEGF also plays an important role in regulating the proliferation and migration of endothelial cells (Bernatchez et al., 1999[3]; Olsson et al., 2006[26]). In order to investigate the possible effects of increased amount of VEGF, caused by exo-OVCAR-3, on proliferation and migration of endothelial cells, HUVECs were incubated with exo-OVCAR-3 (100 µg/ml) for 24 h and 48 h. Wound healing assay was performed to monitor cell migration. We found that HUVECs stimulated with 100 µg/ml exo-OVCAR-3 can migrate significantly faster than HUVECs treated with vehicle control PBS (Figure 4A, B(Fig. 4)). We also showed that exo-OVCAR-3 treatment (100 µg/ml) led to a significant increase in the proliferation rate of HUVECs in a time-dependent manner (Figure 4C(Fig. 4)).

For more results see the Supplementary data.

## Discussion

Cell-to-cell communication is a dynamic process that has an important hand in maintenance of tissue homeostasis. Recent studies have shown that exosomes discharged by various cells partake in this process. Since exosomes represent a protective and enriched source of shuttle coding and non-coding RNAs, they supply the exchange of data between cells (Camussi et al., 2011[5]; Pakravan et al., 2017[27]). Different types of cells including tumor cells can influence their neighbor cells epigenetically by releasing exosomes (Camussi et al., 2013[6]). Indeed, tumor tissue is a mixture of different sorts of cells, where tumor cells are surrounded by tumor-associated stroma cells including vascular endothelial cells, immune-inflammatory cells, mesenchymal stromal cells, and cancer-associated fibroblasts (Plava et al., 2019[28]). The interactions between malignant cells and their microenvironment have been broadly identified (Gouirand et al., 2018[12]; Hall et al., 2007[13]). 

By tumor development and metastasis, the development of blood vessels in the tumor mass is triggered. Actually, the tumor microenvironment takes a center stage in the angiogenic process (Kalluri and Zeisberg, 2006[18]). Tumorigenesis is a convoluted process which is a reflection of various alterations transforming normal cells into exceedingly malignant ones. However, tumor development cannot be simply specified by existence of malignant tumor cells. In other words, different cells and the extracellular matrix (ECM) of the tumor tissue influence the features (Hanahan and Weinberg, 2000[15]).

Angiogenesis, formation of new blood vessels, is one of the key processes in tumor migration. The extracellular matrix can regulate the function of vascular endothelial cells (Nyberg et al., 2005[25]). VEGF is a chief proangiogenic factor that triggers expansion, development, and relocation of endothelial cells; it also increases vascular permeability. VEGF contributes to tumor development and metastasis by increasing tumor-related angiogenesis (Ferrara et al., 2003[11]). Recent studies have revealed that tumor-derived exosomes have promoting effects on VEGF expression in endothelial cells (Sun et al., 2017[31]). Similarly, some studies indicated that the NF-kB pathway is capable of inducing and enhancing the expression of VEGF (Bancroft et al., 2001[1]; Hicklin and Ellis, 2005[17]). In this regard, we initially investigated the effects of exo-OVCAR-3 on VEGF signaling in endothelial cells and found a positive relationship between exo-OVCAR-3 and VEGF expression and secretion levels (Figure 2B(Fig. 2) and Figure 3A, B(Fig. 3)). We also observed that exo-OVCAR-3 treatment leads to a significant and time-dependent increase in the NF-κB mRNA expression level (Figure 2A(Fig. 2)). Consistently, we also observed a significant promoting effect of exo-OVCAR-3 on the proliferative and migratory capability of endothelial cells in a time-dependent manner (Figure 4A-C(Fig. 4)), implying the significance of NF-κB and VEGF expression in endothelial cell proliferation and migration. 

Taken together, we concluded that exosomes derived from ovarian epithelial cancer cells are able to up-regulate VEGF signaling in endothelial cells. What made this study highlighted was to survey the impacts of ovarian cancer cell-derived exosomes on behavior of endothelial cells, especially pertinent to the proliferation and migration. Further knowledge about the mechanisms and functions of cancer-derived exosomes can broaden the horizons toward finding promising novel treatment for ovarian cancer which modulates the cell-to-cell communication in tumor microenvironment.

## Acknowledgements

This work was supported by Tarbiat Modares University and Iran National Science Foundation (INSF) (Grant No. 96008647).

## Conflict of interest

The authors declare that they have no conflict of interest.

## Supplementary Material

Supplementary data

## References

[1] Bancroft CC, Chen Z, Dong G, Sunwoo JB, Yeh N, Park C (2001). Coexpression of proangiogenic factors IL-8 and VEGF by human head and neck squamous cell carcinoma involves coactivation by MEK-MAPK and IKK-NF-κB signal pathways. Clin Cancer Res.

[2] Banerjee S, Kaye SB (2013). New strategies in the treatment of ovarian cancer: current clinical perspectives and future potential. Clin Cancer Res.

[3] Bernatchez PN, Soker S, Sirois MG (1999). Vascular endothelial growth factor effect on endothelial cell proliferation, migration, and platelet-activating factor synthesis is Flk-1-dependent. J Biol Chem.

[4] Borgström P, Hillan KJ, Sriramarao P, Ferrara N (1996). Complete inhibition of angiogenesis and growth of microtumors by anti-vascular endothelial growth factor neutralizing antibody: novel concepts of angiostatic therapy from intravital videomicroscopy. Cancer Res.

[5] Camussi G, Deregibus MC, Bruno S, Grange C, Fonsato V, Tetta C (2011). Exosome/microvesicle-mediated epigenetic reprogramming of cells. Am J Cancer Res.

[6] Camussi G, Deregibus MC, Tetta C (2013). Tumor-derived microvesicles and the cancer microenvironment. Curr Mol Med.

[7] Chowdhury R, Webber JP, Gurney M, Mason MD, Tabi Z, Clayton A (2015). Cancer exosomes trigger mesenchymal stem cell differentiation into pro-angiogenic and pro-invasive myofibroblasts. Oncotarget.

[8] Dinkins MB, Dasgupta S, Wang G, Zhu G, Bieberich E (2014). Exosome reduction in vivo is associated with lower amyloid plaque load in the 5XFAD mouse model of Alzheimer's disease. Neurobiol Aging.

[9] Dvorak H, Nagy J, Feng D, Brown L, Dvorak A (1999). Vascular permeability factor/vascular endothelial growth factor and the significance of microvascular hyperpermeability in angiogenesis. Curr Top Microbiol Immunol.

[10] Egeblad M, Nakasone ES, Werb Z (2010). Tumors as organs: complex tissues that interface with the entire organism. Dev Cell.

[11] Ferrara N, Gerber H-P, LeCouter J (2003). The biology of VEGF and its receptors. Nat Med.

[12] Gouirand V, Guillaumond F, Vasseur S (2018). Influence of the tumor microenvironment on cancer cells metabolic reprogramming. Front Oncol.

[13] Hall B, Andreeff M, Marini F (2007). The participation of mesenchymal stem cells in tumor stroma formation and their application as targeted-gene delivery vehicles. Handb Exp Pharmacol.

[14] Hanahan D, Weinberg RA (2011). Hallmarks of cancer: the next generation. Cell.

[15] Hanahan D, Weinberg RA (2000). The hallmarks of cancer. Cell.

[16] Hayat Nosaeid M, Mahdian R, Jamali S, Maryami F, Babashah S, Maryami F (2009). Validation and comparison of two quantitative real-time PCR assays for direct detection of DMD/BMD carriers. Clin Biochem.

[17] Hicklin DJ, Ellis LM (2005). Role of the vascular endothelial growth factor pathway in tumor growth and angiogenesis. J Clin Oncol.

[18] Kalluri R, Zeisberg M (2006). Fibroblasts in cancer. Nat Rev Cancer.

[19] Lee J-K, Park S-R, Jung B-K, Jeon Y-K, Lee Y-S, Kim M-K (2013). Exosomes derived from mesenchymal stem cells suppress angiogenesis by down-regulating VEGF expression in breast cancer cells. PloS One.

[20] Liang B, Peng P, Chen S, Li L, Zhang M, Cao D (2013). Characterization and proteomic analysis of ovarian cancer-derived exosomes. J Proteomics.

[21] Livak KJ, Schmittgen TD (2001). Analysis of relative gene expression data using real-time quantitative PCR and the 2(-Delta Delta C(T)) method. Methods.

[22] Mariappan L, Jiang XY, Jackson J, Drew Y (2017). Emerging treatment options for ovarian cancer: focus on rucaparib. Int J Womens Health.

[23] McMahon G (2000). VEGF receptor signaling in tumor angiogenesis. Oncologist.

[24] Motavaf M, Pakravan K, Babashah S, Malekvandfard F, Masoumi M, Sadeghizadeh M (2016). Therapeutic application of mesenchymal stem cell-derived exosomes: A promising cell-free therapeutic strategy in regenerative medicine. Cell Mol Biol (Noisy-le-grand).

[25] Nyberg P, Xie L, Kalluri R (2005). Endogenous inhibitors of angiogenesis. Cancer Res.

[26] Olsson A-K, Dimberg A, Kreuger J, Claesson-Welsh L (2006). VEGF receptor signalling? In control of vascular function. Nat Rev Mol Cell Biol.

[27] Pakravan K, Babashah S, Sadeghizadeh M, Mowla SJ, Mossahebi-Mohammadi M, Ataei F (2017). MicroRNA-100 shuttled by mesenchymal stem cell-derived exosomes suppresses in vitro angiogenesis through modulating the mTOR/HIF-1alpha/VEGF signaling axis in breast cancer cells. Cell Oncol (Dordr).

[28] Plava J, Cihova M, Burikova M, Matuskova M, Kucerova L, Miklikova S (2019). Recent advances in understanding tumor stroma-mediated chemoresistance in breast cancer. Mol Cancer.

[29] Shahi PK, Pineda IF (2008). Tumoral angiogenesis: review of the literature. Cancer Invest.

[30] Sharma S, Zuñiga F, Rice GE, Perrin LC, Hooper JD, Salomon C (2017). Tumor-derived exosomes in ovarian cancer–liquid biopsies for early detection and real-time monitoring of cancer progression. Oncotarget.

[31] Sun X, Ma X, Wang J, Zhao Y, Wang Y, Bihl JC (2017). Glioma stem cells-derived exosomes promote the angiogenic ability of endothelial cells through miR-21/VEGF signal. Oncotarget.

[32] Wei W, Dizon D, Vathipadiekal V, Birrer M (2013). Ovarian cancer: genomic analysis. Ann Oncol.

[33] Welti J, Loges S, Dimmeler S, Carmeliet P (2013). Recent molecular discoveries in angiogenesis and antiangiogenic therapies in cancer. J Clin Invest.

[34] Wu L, Zhang X, Zhang B, Shi H, Yuan X, Sun Y (2016). Exosomes derived from gastric cancer cells activate NF-κB pathway in macrophages to promote cancer progression. Tumor Biol.

[35] Wu Y, Hooper AT, Zhong Z, Witte L, Bohlen P, Rafii S (2006). The vascular endothelial growth factor receptor (VEGFR‐1) supports growth and survival of human breast carcinoma. Int J Cancer.

