# Supplementary material for: The effects of ovarian cancer cell-derived exosomes on vascular endothelial growth factor expression in endothelial cells
Source: EXCLI J. 2019 Oct 9;18:899–907. doi: 10.17179/excli2019-1800 (PMC6806135; doi:10.17179/excli2019-1800)
Supplement: Supplementary data [file EXCLI-18-899-s-001.pdf]

*EXCLI Journal 2019;18:899-907 – ISSN 1611-2156*
*Received: September 08, 2019, accepted: October 08, 2019, published: October 09, 2019*

## Supplementary data to:

# THE EFFECTS OF OVARIAN CANCER CELL-DERIVED EXOSOMES ON VASCULAR ENDOTHELIAL GROWTH FACTOR EXPRESSION IN ENDOTHELIAL CELLS

Mohammad Ghorbanian[1], Sadegh Babashah[1,*], Farangis Ataei[2]

[1] Department of Molecular Genetics, Faculty of Biological Sciences, Tarbiat Modares University, Tehran, Iran
[2] Department of Biochemistry, Faculty of Biological Sciences, Tarbiat Modares University, Tehran, Iran

* **Corresponding author:** Sadegh Babashah, PhD, Department of Molecular Genetics, Faculty of Biological Sciences, Tarbiat Modares University, P.O. Box: 14115-154, Tehran, Iran, Tel.: +98 21 8288 4468, Fax: +98 21 8288 4717,
E-mail: sadegh.babashah@gmail.com; babashah@modares.ac.ir

http://dx.doi.org/10.17179/excli2019-1800

This is an Open Access article distributed under the terms of the Creative Commons Attribution License (http://creativecommons.org/licenses/by/4.0/).

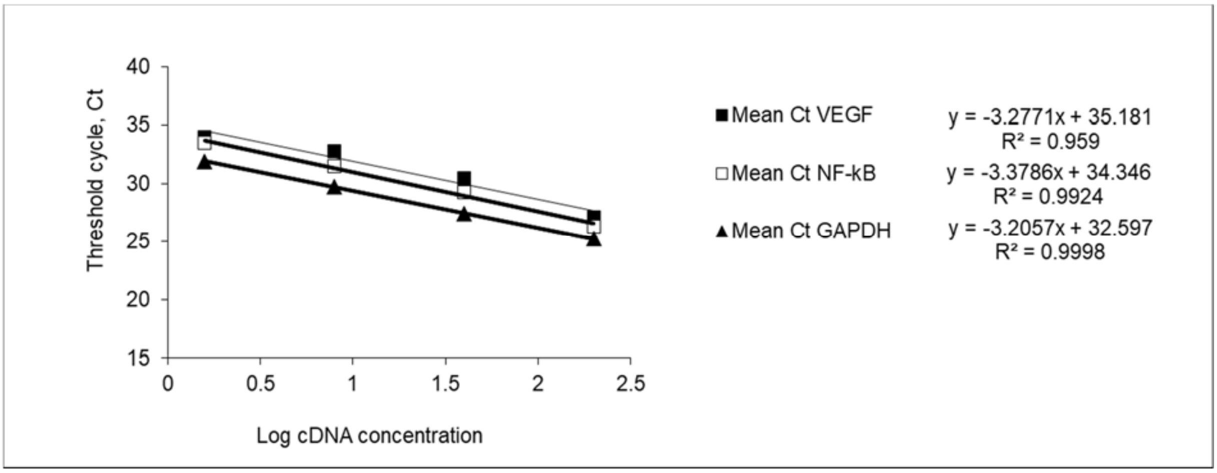


**Supplementary Figure 1: RT-qPCR efficiencies.** To determine the amplification efficiency, standard curves via plotting the logarithmic amount of serially diluted cDNA input against the corresponding Ct values was exploited. The efficiency (E) of RT-qPCR was calculated according to the slope of the standard curve and the following equation: $E = 10^{[-1/slope]}$. All slopes were approximately equal with high linear correlation.

EXCLI Journal 2019;18:899-907 – ISSN 1611-2156
Received: September 08, 2019, accepted: October 08, 2019, published: October 09, 2019

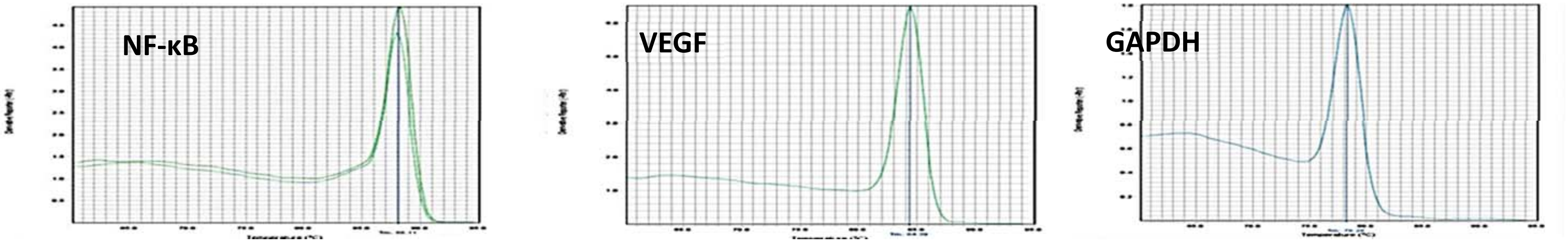


**Supplementary Figure 2: Uniqueness and specificity of the RT-qPCR products.** Dissociation curve analysis performed on PCR products obtained from amplification reactions for NF-κB, VEGF, and GAPDH. The curves featured by a single and sharp peak at expected Tm.

VEGF, Vascular endothelial growth factor; NF-κB, Nuclear factor kappa-light-chain-enhancer of activated B cells; GAPDH, Glyceraldehyde 3-phosphate dehydrogenase

*EXCLI Journal 2019;18:899-907 – ISSN 1611-2156*
*Received: September 08, 2019, accepted: October 08, 2019, published: October 09, 2019*

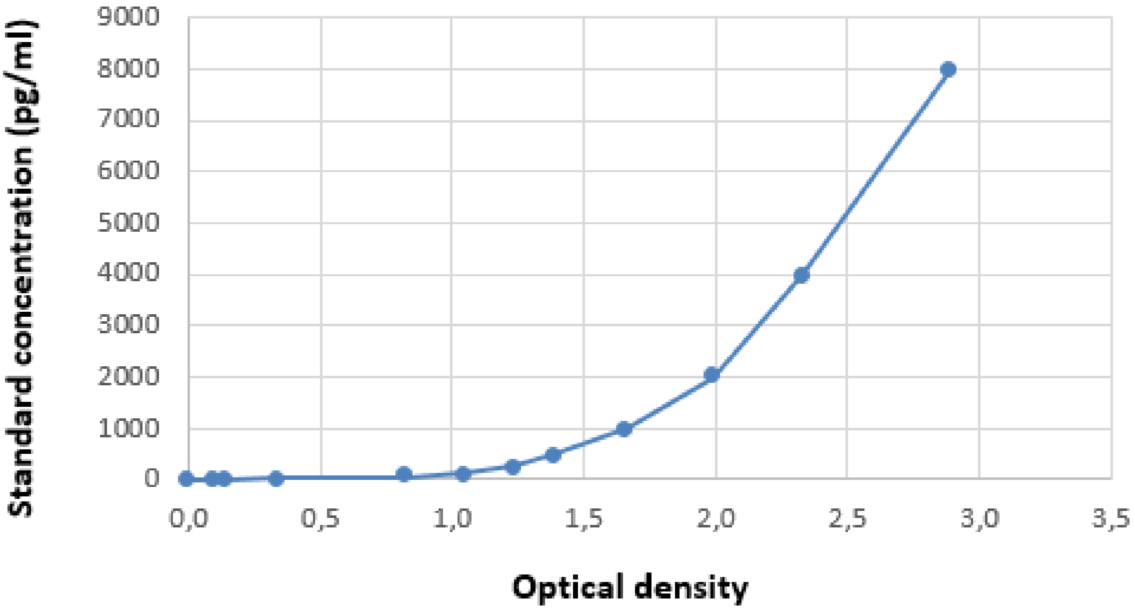


**Supplementary Figure 3: ELISA standard curve**

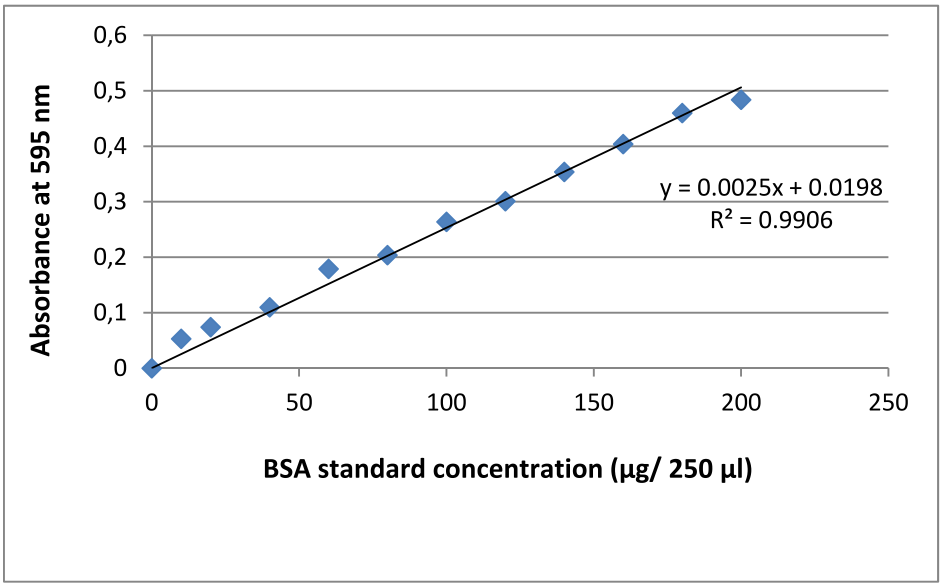


**Supplementary Figure 4: Bovine Serum Albumin (BSA) standard curve**; in equation (x) stands for sample concentration and (y) stands for absorbance at 595 nm. The data are fit with linear regression by the line y= 0.0025x + 0.0198 with $R^2$ value of 0.9906.

EXCLI Journal 2019;18:899-907 – ISSN 1611-2156
Received: September 08, 2019, accepted: October 08, 2019, published: October 09, 2019

**Supplementary Table 1:** Slope and efficiencies of standard curves for each primer set

| Gene | Slope | Efficiency |
|---|---|---|
| VEGF | -3.2771 | 1.02 |
| NF-κB | -3.3786 | 0.98 |
| GAPDH | -3.2057 | 1.05 |

**Supplementary Table 2: Enzyme-linked immunosorbent assay (ELISA) data reduction for plotting a standard curve. Table lists data of standard concentrations, absorbance values, and corrected absorbance value.** Absorbance of standards are corrected by subtracting OD of blank well (0.174).

| | Concentration (pg/ml) | OD1 | OD2 | OD Average | OD Corrected |
|---|---|---|---|---|---|
| Blank | 0.0 | 0.172 | 0.176 | 0.174 | 0.000 |
| Standard 1 | 7.8 | 0.275 | 0.276 | 0.275 | 0.101 |
| Standard 2 | 15.6 | 0.313 | 0.314 | 0.314 | 0.140 |
| Standard 3 | 31.2 | 0.513 | 0.517 | 0.515 | 0.341 |
| Standard 4 | 62.5 | 1.013 | 0.991 | 1.002 | 0.828 |
| Standard 5 | 125.0 | 1.233 | 1.215 | 1.224 | 1.050 |
| Standard 6 | 250.0 | 1.429 | 1.389 | 1.409 | 1.235 |
| Standard 7 | 500.0 | 1.592 | 1.539 | 1.566 | 1.392 |
| Standard 8 | 1000 | 1.849 | 1.822 | 1.835 | 1.661 |
| Standard 9 | 2000 | 2.189 | 2.145 | 2.167 | 1.993 |
| Standard 10 | 4000 | 2.569 | 2.442 | 2.506 | 2.332 |
| Standard 11 | 8000 | 3.163 | 2.972 | 3.067 | 2.893 |

**Supplementary Table 3: Standards preparation scheme and resulted absorbance.** Absorbance at 595 nm (after subtraction of blank absorbance (0.238) for eleven standards

| µg/ml | 0 | 10 | 20 | 40 | 60 | 80 | 100 | 120 | 140 | 160 | 180 | 200 | sample |
|---|---|---|---|---|---|---|---|---|---|---|---|---|---|
| Blank | 0.238 | 0.291 | 0.312 | 0.348 | 0.417 | 0.442 | 0.502 | 0.539 | 0.592 | 0.642 | 0.698 | 0.722 | 0.547 |
| OD595 | 0 | 0.053 | 0.074 | 0.11 | 0.179 | 0.204 | 0.264 | 0.301 | 0.354 | 0.404 | 0.46 | 0.484 | 0.309 |
